# Fungal Lignocellulose Utilisation Strategies from a Bioenergetic Perspective: Quantification of Related Functional Traits Using Biocalorimetry

**DOI:** 10.3390/microorganisms10081675

**Published:** 2022-08-19

**Authors:** Hieu Linh Duong, Sven Paufler, Hauke Harms, Dietmar Schlosser, Thomas Maskow

**Affiliations:** 1 Department of Environmental Microbiology, Helmholtz Centre for Environmental Research-UFZ, Permoserstraβe 15, 04318 Leipzig, Germany; 2 Faculty of Engineering, Vietnamese-German University (VGU), Le Lai Street, Hoa Phu Ward, Thủ Dầu Một 7500, Binh Duong, Vietnam

**Keywords:** ascomycete, basidiomycete, biothermodynamics, ecological theory, functional trait, fungal growth, life history strategy, lignocellulose, wheat straw, zygomycete

## Abstract

In the present study, we investigated whether a non-invasive metabolic heat flux analysis could serve the determination of the functional traits in free-living saprotrophic decomposer fungi and aid the prediction of fungal influences on ecosystem processes. For this, seven fungi, including ascomycete, basidiomycete, and zygomycete species, were investigated in a standardised laboratory environment, employing wheat straw as a globally relevant lignocellulosic substrate. Our study demonstrates that biocalorimetry can be employed successfully to determine growth-related fungal activity parameters, such as apparent maximum growth rates (*AMGR*), cultivation times until the observable onset of fungal growth at *AMGR* (*t_AMGR_*), quotients formed from the *AMGR* and *t_AMGR_* (herein referred to as competitive growth potential, *CGP*), and heat yield coefficients (*Y_Q/X_*), the latter indicating the degree of resource investment into fungal biomass versus other functional attributes. These parameters seem suitable to link fungal potentials for biomass production to corresponding ecological strategies employed during resource utilisation, and therefore may be considered as fungal life history traits. A close connection exists between the *CGP* and *Y_Q/X_* values, which suggests an interpretation that relates to fungal life history strategies.

## 1. Introduction

Fungi are the primary decomposers of lignocellulose-based materials such as litter and wood, and thus play a key role in the global carbon cycle [1]. Fungi are further considered as promising biocatalysts for the conversion of lignocellulosic by-products from agriculture and forestry into various useful products, e.g., in biorefinery applications, and they enable the use of lignocellulosic residues for nutritional and bioremediation purposes [2,3].

Lignocellulosic materials represent highly heterogeneous fungal substrates with respect to both their chemical composition and spatial organisation, which largely influences the dynamics of fungal degrader communities and related ecological successions [4,5,6,7]. Clearly, the understanding of the unifying principles that govern fungal community organisation and the associated possible prediction of community function [1,5,8,9] can be expected to support the sustainable use of ecosystems through the maintenance, facilitation, and/or promotion of ecosystem services brought about by fungal organisms. Moreover, the understanding and possible controlling of fungal community dynamics and functions are also highly relevant for industrial-scale biotechnological processes aiming at the valorisation of lignocellulosic and other agricultural wastes, especially considering that such processes ideally working under non-sterile conditions are unfavourable for actual pure culture scenarios [10,11,12].

Fungal functional traits, which may be defined as “measurable properties of organisms that are used comparatively across individuals and influence an organism’s performance or fitness” according to Crowther et al. [8], have been proven to be useful tools for the prediction of fungal influences on ecosystem processes [1,7,9,13,14]. Measurable functional traits related to fungal lifestyles involve, e.g., growth, respiration, and decomposition rates; the expression of catabolic enzymes; the production of functional compounds such as siderophores, chelators, hydrophobins, or antibiotics; and the formation and characteristics of spores/propagules [1,7,14]. In order to aid functional assignments and the ecological interpretation of environmental studies, traits of fungi and fungus-like organisms can nowadays be retrieved from databases such as FungalTraits, FUNGuild, and FunFun [9,15].

Calorimetry is an excellent tool for tracking fungal growth as it provides real-time metabolic information, does not affect growth and product formation processes, and delivers thermodynamic state variables for the biothermodynamic characterisation and prediction of fungal activities. Examples of the successful application of calorimetry are the quantification of growth processes [16,17], the analysis of the fungal degradation of wooden building materials [18,19], the detection of maize seed spoilage [20], and the quantification of carotenoid production [21]. In a previous paper, we applied the non-invasive measurement of metabolic heat fluxes to monitor fungal activity during the colonisation of a solid lignocellulosic substrate [2]. For both the fast-growing, cellulose-utilising ascomycetous mould *Stachybotrys chlorohalonata* and the comparatively slow-growing, litter-decaying basidiomycete *Stropharia rugosoannulata* (the latter causing a white-rot decay type of lignocellulose), the fungal biomass yield was found to be strongly correlated with the released metabolic heat. The corresponding species-specific heat yield coefficients (i.e., the metabolic heat released per increase of fungal biomass unit; *Y_Q/X_*) pointed to different fungal life history strategies during resource utilisation: in *S. chlorohalonata*, it suggested a prominent resource investment into growth, and in *S. rugosoannulata*, it suggested resource investment into further functional attributes at the expense of fungal biomass formation [2].

**Figure 1:**
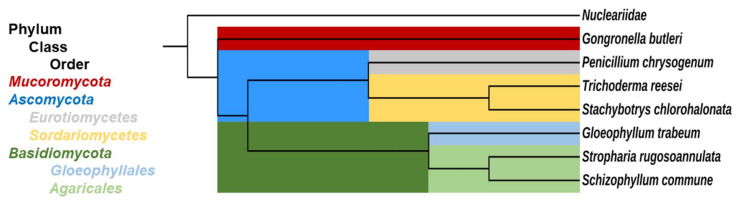
Phylogenetic tree displaying the relationship of the fungal strains used in this study, based on NCBI taxonomy data [45]. The tree was generated in phyloT (https://phylot.biobyte.de/) (accessed on 18 November 2021) and visualised with iTOL [46]. The family Nucleariidae, a group of amoebae near the origin of the animal-fungal divergence [47], is shown as an outgroup.

The aforementioned results encouraged us to investigate whether and how convenient, non-invasive biocalorimetric measurements could serve to quantify and perhaps more precisely define [1,14] functional traits in free-living saprotrophic lignocellulose decomposer fungi. In particular, we were interested in traits related to fungal growth strategies and the extent of resource investment into fungal biomass formation. Following a previous definition, we considered a fungal trait as a characteristic of an individual fungus that could be measured under standardised growth conditions and compared across individuals [1]. For the purposes of our study, we used wheat straw as a proxy for a lignocellulosic substrate of global relevance, which also possesses a great potential for biotechnological valorisation, e.g., for use in chemical building blocks, other raw materials, bioenergy, or compost [2]. Measurements of the metabolic heat fluxes caused by fungal substrate colonisation were linked to conventional invasive determinations of fungal activity (growth, substrate degradation) parameters [2] in order to characterise the growth and substrate utilisation strategies of the selected fungal model species. These comprised ascomycete, basidiomycete, and zygomycete saprotrophs that are known to grow on lignocellulose, which were newly investigated within the present study. We also included previously investigated ascomycete (*S. chlorohalonata*) and basidiomycete species (*S. rugosoannulata*) [2]; thereby considering different types of lignocellulose decay altogether (Table 1). In addition to the last-named species, corresponding fungal representatives of different ecophysiological groups include the wood-decay basidiomycetes *Gloeophyllum trabeum* and *Schizophyllum commune*, the ascomycetes *Trichoderma reesei* and *Penicillium chrysogenum*, and the zygomycete *Gongronella butleri*. The basidiomycete *Gloeophyllum trabeum* was chosen to represent brown-rot fungi, which oxidatively degrade particular wood cell wall components with the help of extracellularly generated reactive oxygen species (ROS), resulting in the severe loss of wood strength through the utilisation of its polysaccharide components for growth, while leaving modified lignin as a polymeric brown residue [4,22,23]. *Schizophyllum commune*, another wood-rotting basidiomycete, can be considered as an intermediate between brown-rot and white-rot fungi as based on its lignocellulose-acting enzyme inventory, which lacks typical lignin-modifying class II peroxidases (such as brown-rot fungi) but possesses enzymes that act on crystalline cellulose (such as white-rot fungi) [23]. *S. commune* degrades plant lignocellulose via the action of ROS, such as hydroxyl radicals in concert with various polysaccharide-degrading enzymes, and enhances its access to polysaccharides with the help of multiple non-hydrolytic proteins, including lytic polysaccharide monooxygenases (LPMOs) and expansin-like proteins [24]. The soft-rot ascomycete *Trichoderma reesei* is an important and widely exploited workhorse for the production of industrial enzymes, particularly in the production of cellulose- and hemicellulose-degrading enzymes [25,26,27]. There are 9 characterised cellulases, 15 confirmed hemicellulases, and at least 42 genes predicted to encode carbohydrate-active enzymes (CAZy) that have been identified in the genome of *T. reesei* [26,28,29]. Besides *T. reesei*, the environmentally ubiquitous ascomycetous mould *Stachybotrys chlorohalonata* is another soft-rot representative with a known capacity to produce cellulose-degrading enzymes [2,30,31,32]. *Penicillium chrysogenum*, an established producer of the antibiotic penicillin, was also included in the present study. Apart from penicillin production, this fungus is known to produce various lignocellulolytic enzymes, including hemicellulases such as acetyl xylan esterase [33], arabinofuranosidase [34], xylanase [35,36,37], and mannanase [36]. Under the induction of lignocellulose, *P. chrysogenum* was shown to secrete complete cellulases and numerous hemicellulases [38]. In addition to ascomycetes and basidiomycetes, the mucoromycetous zygomycete *Gongronella butleri* [39,40] was also investigated. Zygomycetes are increasingly being used for the production of fermented foods and a variety of metabolic products such as lactic acid, fumaric acid, and ethanol from a wide range of feedstocks [41]. Their biomass has long been recognised as a valuable source of lipids, proteins with superior amino acid composition, and chitosan, the latter being produced in high amounts by *G. butleri* [41,42]. The phylogenetic relationships among the fungal strains assessed within the present study (Table 1) are illustrated in Figure 1.

## 2. Materials and Methods

### 2.1. Chemicals

All chemicals were of analytical grade (gradient grade in the case of the chromatography solvents), unless stated otherwise. 2,2′-Azinobis (3-ethylbenzothiazoline-6-sulfonic acid) (ABTS, purity > 98%) was obtained from AppliChem (Darmstadt, Germany). All other chemicals were purchased from Merck, Sigma-Aldrich, and Th. Geyer GmbH (Renningen, Germany).

### 2.2. Source and Maintenance of Fungal Strains

*Gloeophyllum trabeum* (DSM 1398) and *Penicillium chrysogenum* (DSM 848) (Figure 1, Table 1) (also available from the DSMZ German Collection of Microorganisms and Cell Cultures; Braunschweig, Germany) were obtained from the strain collection of the Department of Environmental Microbiology at the Helmholtz Centre for Environmental Research-UFZ (Leipzig, Germany). *Gongronella butleri* (DSM 2917), *Schizophyllum commune* (DSM 11223), and *Trichoderma reesei* (DSM 769) (Figure 1, Table 1) were obtained from the German Collection of Microorganisms and Cell Cultures (DSMZ; Braunschweig, Germany). The fungal strains were maintained on 2% (*w*/*v*) of malt extract agar plates (1.5% agar; pH 5.7) at 28 °C in the dark.

### 2.3. Fungal Cultivations on Wheat Straw

The axenic fungal cultivation on wheat straw using polypropylene vials for calorimetric measurements and all other accompanying analyses are comprehensively described in [2]. Briefly, the sterile straw was aseptically inoculated with the respective fungal strains in the calorimetric polypropylene bottles. The vials were equipped with venting membrane screw caps (PTFE membrane with a 25 mm diameter and a 0.2 μm porosity, Whatman/GE Healthcare, Germany). For the inoculation with fungi, agar plugs (derived from the edges of the fungal colonies grown on malt agar plates as described above) were homogenised in 2% of malt extract medium (one agar plug per 1 mL of malt extract medium) with the help of an Ultra-Turrax (Staufen, Germany). Thereafter, 0.5 mL of the resulting fungal suspension was used to inoculate one calorimetric vial, respectively. For the controls, 0.5 mL of sterile malt extract medium (2%) was added to the vials containing wheat straw. The experimental setup differed from our previous work [2] in the following manner: Triplicate vials per fungus and triplicate sterile controls (non-inoculated), incubated in the isothermal microcalorimeter (see below) were harvested for subsequent analyses after 20 days. In addition, the triplicate controls were also harvested at the beginning of the experiments. After harvesting, all vials were stored frozen at −20 °C until the analysis.

### 2.4. Isothermal Microcalorimetry

Heat production rates were determined against 3 mL of sterile tap water as a reference by an isothermal microcalorimetry technique at 28.000 ± 0.001 °C using an MC CAL isothermal microcalorimeter (C3 Prozess- und Analysentechnik GmbH, Haar b. München, Germany) [2]. This amount of water was chosen to ensure that the heat capacity of the sample and the reference were approximately equal. The instrument, the limit of detection, and the performed calibration procedure using Joule heating, is described in [21]. The calorimetric vials (40 mL volume; [2]) were filled with 3 g of sample (0.5 g of dry wheat straw + 2 mL of tap water + 0.5 mL of fungal inoculum) as described in [2]. In order to ensure a sufficient oxygen concentration and to prevent the accumulation of produced CO_2_ in the calorimetric vials, these vials were aseptically opened from a quarter-minute to a half-minute on each day of the working week (i.e., from Monday to Friday). The thermal disturbance caused by the opening was not considered in the data evaluation. The heat production rate during the disturbance was linearly interpolated from the signals before and after the disturbance [2]. The software OriginPro 2020 (OriginLab Corp., Northampton, MA, USA) was used to evaluate the calorimetric signals.

### 2.5. Further Analytical Procedures

All further analytical methods aimed at the substrate characteristics, fungal activity, and corresponding sample preparations were carried out as previously described [2]. These included the determinations of the: (i) total dry masses; (ii) fungal biomasses based on ergosterol content; (iii) lignin contents using Fourier transform mid-infrared (FT-MIR) spectroscopy; and (iv) total reducing sugars [2]. Solids that remained after the aqueous extraction of samples, as described in [2], were dried on pre-weighted filter papers (Whatman No. 1, Maidstone, UK) at 50 °C for 48 h.

### 2.6. Statistical Analyses

Outliers among triplicate data sets were identified using a Dean–Dixon test [48]. The softwares OriginPro 2020 and R (version 4.1.0) were used to check data for normality according to the Kolmogorov–Smirnov and Shapiro–Wilk tests, respectively. The student’s *t*-tests were performed using Microsoft^®^ Excel^®^ 2013 (version 15.0.5327.1000). The linear and non-linear correlation analyses were performed using OriginPro 2020, as outlined in the paper.

## 3. Results

### 3.1. Substrate Decomposition and Fungal Growth during Fungal Wheat Straw Colonisation

**Figure 2:**
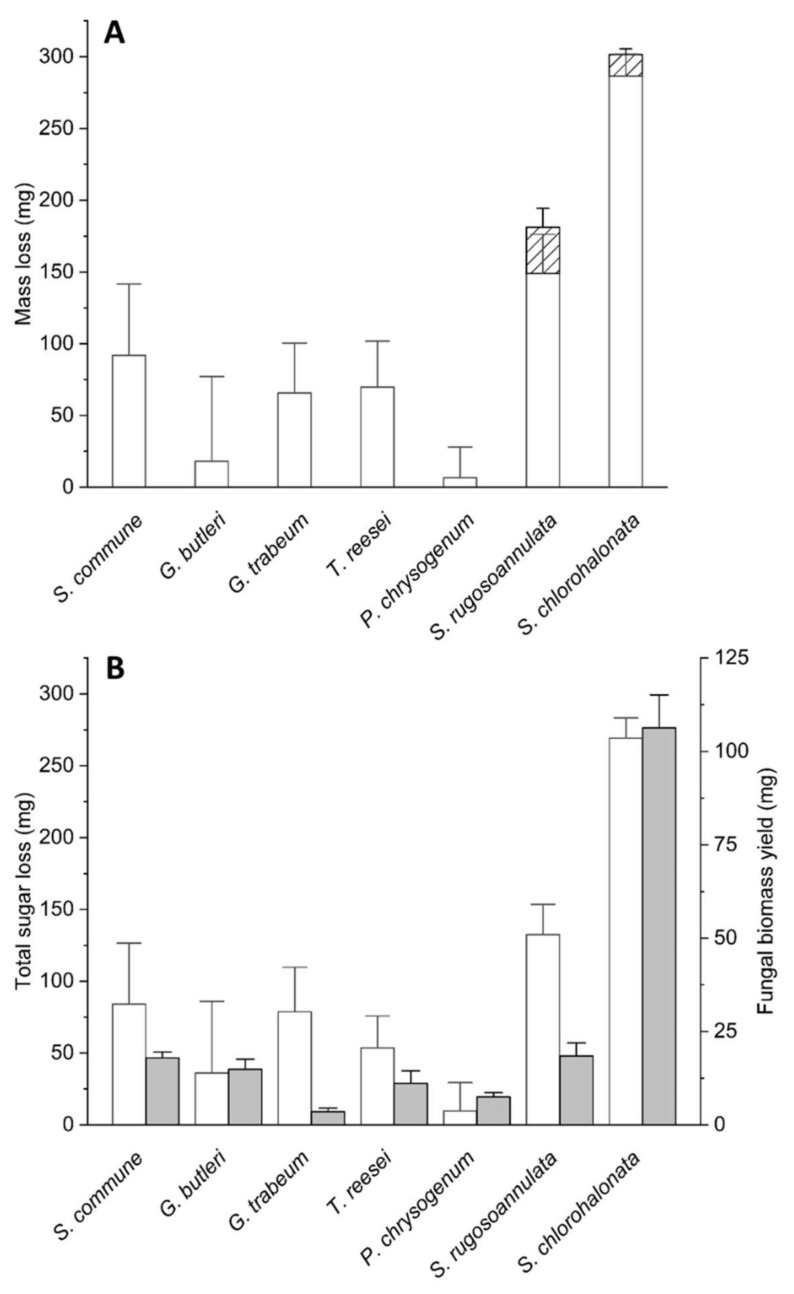
(**A**) Losses in the lignin contents (hatched bars) and total substrate dry mass losses (sum of both the white and hatched bars, respectively); (**B**) losses of total sugars (white bars) and biomass yields (grey bars) of the investigated fungal wheat straw cultures. The symbols and error bars represent the means and standard deviations (calculated according to the Gaussian error propagation rules) for triplicate cultures, respectively. Data for *S. rugosoannulata* and *S. chlorohalonata* were already reported before [2], and re-arranged to meet the requirements of the current Figure 2. The data correspond to total cultivation periods of either 32 (in the case of *S. rugosoannulata* and *S. chlorohalonata*) or 20 days (all other fungi). The underlying lignin contents, dry masses, total sugars in the solids remaining after aqueous extraction, total water-extractable sugars, and fungal biomasses in terms of absolute quantities are compiled in Tables S1 and S2 in the Supplementary Materials.

Figure 2 depicts the effects of fungal colonisation on lignin and substrate dry mass loss, loss of total sugars (expressed as the sum of total sugars of solids remaining after aqueous extraction and total water-extractable sugars, respectively), and fungal biomass yields (always calculated as the difference between the corresponding initial and final amounts, respectively) at the end of the respective cultivation periods. No significant losses were observed in the total dry masses, lignin, and sugar contents in the abiotic controls throughout the entire incubation period of experiments (*p* > 0.05 in the two-sample Student’s *t*-tests), and fungal growth in the controls could not be detected. The underlying lignin contents, total dry masses, total amount of sugars in the solids remaining after aqueous extraction, total water-extractable sugars, and fungal biomasses in terms of absolute quantities are compiled in Supplementary Tables S1 and S2. Substrate dry masses were calculated as the differences between total dry masses (Table S1) and fungal biomasses (Table S2), respectively.

We observed substrate dry mass losses in the range of approximately 12–17% (in relation to the corresponding initial values) for *T. reesei*, *G. trabeum*, and *S. commune* (Figure 2A, Table S1). Only minor substrate mass losses of about 1–3% could be recorded for *G. butleri* and *P. chrysogenum* (Figure 2A, Table S1). The substrate dry mass losses observed with *S. rugosoannulata* and *S. chlorohalonata* were the most prominent, where a range of about 29–58% substrate mass reduction was detected (Figure 2A, Table S1). The substrate dry mass loss was accompanied by a substantial delignification in the cultures of the white-rot basidiomycete *S. rugosoannulata*, with lignin removal accounting for the observed total dry mass loss of about 22% and a lignin loss of about 43% of the initial lignin content [2] (Figure 2A, Table S2). Furthermore, the soft-rot ascomycete *S. chlorohalonata* showed some minor delignification [2], whereas all other tested strains did not noticeably delignify the wheat straw substrate (Figure 2A, Table S2).

Figure 2B displays the removal of total sugars based on the sum of the total sugars in the solids remaining after aqueous extraction and total water-extractable sugars, respectively; the latter sugar fraction mostly accounted for less than 10% (less than 17% in the case of *S. chlorohalonata*) of the sum of total sugars (Table S2). The most efficient sugar removal was observed in *S. chlorohalonata,* followed by *S. rugosoannulata*, *S. commune*, *G. trabeum*, *T. reesei*, *G. butleri*, and *P. chrysogenum* (Figure 2B, Table S2).

The fungal biomass yield coefficients (*Y_X/S_*), which were calculated from the respective fungal biomass yields (*X*) and total sugars consumed (*S*: in consideration of the sum of total sugars of solids and total water-extractable sugars as described above), displayed considerable differences among the investigated fungal strains (Table 2). Strikingly low values of 0.05 and 0.14 were obtained for the brown-rot basidiomycete *G. trabeum* and the litter-inhabiting white-rot basidiomycete *S. rugosoannulata*, respectively. The ascomycetous mould *P. chrysogenum* yielded the highest *Y_X/S_* observed for the investigated fungi (0.77), albeit this value was associated with a considerable error, as could be deduced from the standard deviation being about twice as high as the mean value. Intermediate values ranging from 0.21 (*S. commune*, *T. reesei*) to 0.41 (*G. butleri*) were derived from the other investigated fungal strains (Table 2).

### 3.2. Lignocellulose Decomposition Process and Fungal Activity Characterisation Involving Metabolic Heat Production Analysis

**Figure 3:**
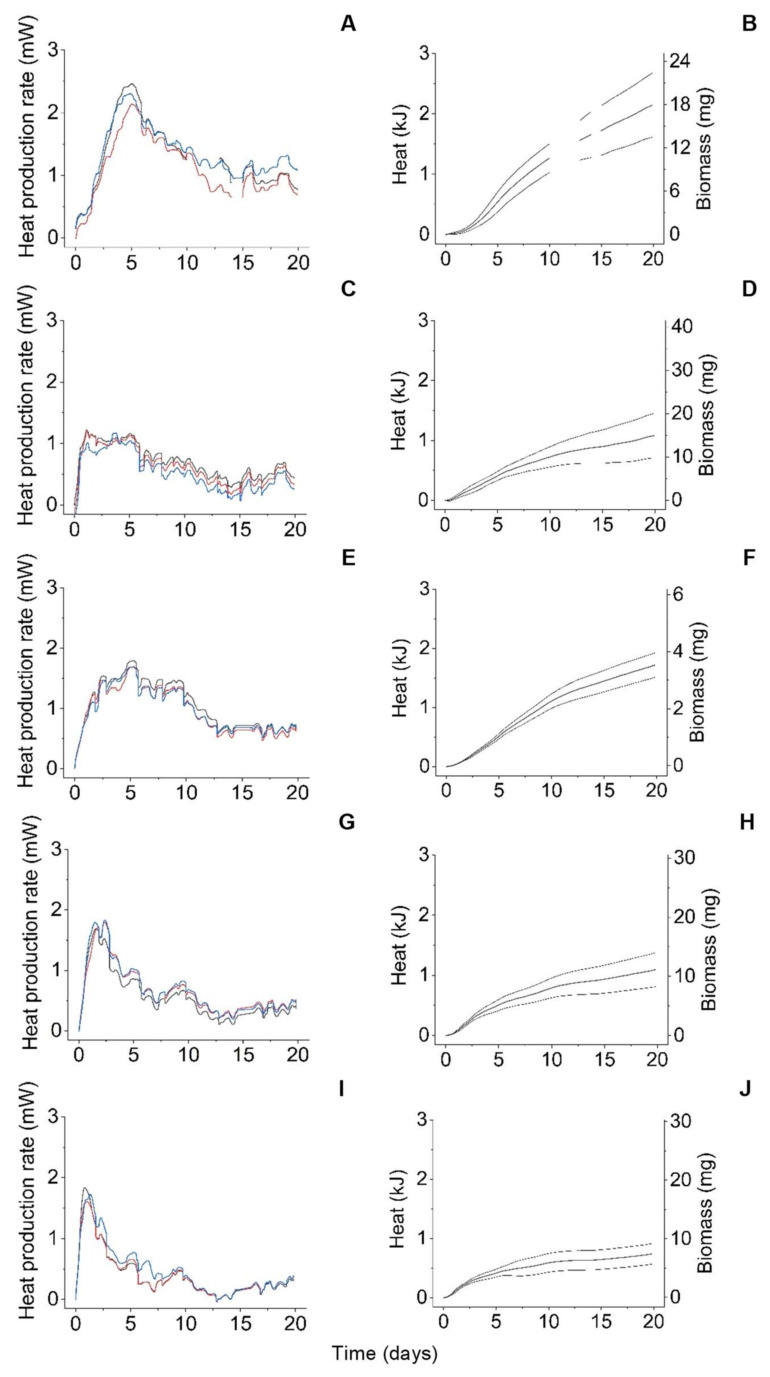
Metabolic heat production rate versus time (**A**,**C**,**E**,**G**,**I**), and heat (integral of the heat production rate) and fungal biomass increase (i.e., biomass yield) versus time (**B**,**D**,**F**,**H**,**J**) during the growths of *S. commune* (**A**,**B**), *G. butleri* (**C**,**D**), *G. trabeum* (**E**,**F**), *T. reesei* (**G**,**H**), and *P. chrysogenum* (**I**,**J**) on wheat straw. Triplicate traces shown in black, blue and red colour in A,C,E,G,I, respectively, always stem from triplicate fungal cultures. The bold lines in B,D,F,H,J are the means of the triplicate fungal cultures, and the thin dotted lines denote the corresponding upper and lower 95% confidence limits, respectively (not calculated for the occasional time periods of the suspended recording of the heat production rate due to technical reasons, as can be seen in Figure 3A; this led to gaps in the traces shown in Figure 3B).

Figure 3 depicts the time courses of the metabolic heat production rate (Figure 3A,C,E,G,I) and the associated metabolic heat (*Q*: the integral of the respective heat production rate over time, Figure 3B,D,F,H,J) for the wheat straw cultures of *S. commune*, *G. butleri*, *G. trabeum*, *T. reesei*, and *P. chrysogenum*. All observed heat production rates had essentially become stagnant after 20 days of cultivation (Figure 3A,C,E,G,I), thus ensuring comparability with the corresponding data of *S. chlorohalonata* and *S. rugosoannulata* published earlier [2]. The unusually high signal fluctuations (approx. 0.2 mW) within a period of approx. one day can be attributed to either daily oscillations in the samples or an instrument error. Information from the manufacturer (thermal detection limit of 0.08 mW) and our own preliminary work (long term signal noise of 0.02 mW, [21]) determined an error resulting from the calorimetric instrument to be unlikely.

The metabolic heat yield coefficients (*Y_Q/X_*) of the investigated fungal strains, which represent the quotients of the cumulative metabolic heat released (*Q*) divided by the corresponding fungal biomass yield (*X*; Figure 2B), respectively, are listed in Table 2. The highest values (in descending order) were observed for the lignocellulose decaying basidiomycetes *G. trabeum*, *S. rugosoannulata*, and *S. commune*. The other investigated fungi followed the rank order *P. chrysogenum* > *T. reesei* > *G. butleri* > *S. chlorohalonata*, with the latter species yielding the lowest value by far (Table 2).

Generally, the *Y_Q/X_* depends on the energy content (e.g., enthalpy of the combustion) of the substrate and on the energy utilisation by the metabolism of the microorganism (in our case, the fungi) [49]. The energy content of the substrates can be determined by a combustion calorimetry technique or can be estimated from the relative degree of reduction $\gamma_{i}$ (Equations (1) and (2)) [49].

(1) $$\Delta_{C}H_{i}^{0}=Q_{0}\cdot \gamma_{i}$$

(2) $$\gamma_{i}=\frac{4\cdot n_{C}+n_{H}-2\cdot n_{O}+3\cdot n_{N}+6\cdot n_{S}}{n_{C}}$$

Here, the combustion enthalpy is related to one C-mol (to illustrate: in the C-mol notation, CH_2_O is written instead of C_6_H_12_O_6_ for glucose). Sandler and Orbey suggested *Q_0_* = −109.04 kJ/C-mol, with an average error of 55.2 kJ/mol (21.3 kJ/C-mol) for 106 different compounds [50]. The symbols *n_C_*, *n_H_*, *n_O_*, *n_N_*, and *n_S_* stand for the numbers of hydrogen, oxygen, nitrogen, and sulfur atoms in the molecule of the substrate, respectively. Lignin, cellulose, and hemicellulose are the main components of straw. Pure lignin has a higher calorific value (−20.4 kJ/g) than pure cellulose (−16.5 kJ/kg) and hemicellulose (−13.9 kJ/kg) [51]. Depending on the data source, the heat of combustion of lignin may vary considerably between −17.0 and −29.2 kJ/g, with an average value of −25.5 kJ/g [52]. Estimating the combustion enthalpy of lignin from its elemental composition using the data of Li et al. [53] and Sameni et al. [54] and taken together with Equations (1) and (2), a combustion enthalpy of −24.9 ± 2.3 kJ/g (−487.9 kJ/C-mol) was calculated, which was used in the following calculations. The heat of the combustion of cellulose depends on the source of this compound (e.g., cotton linters, wood pulp), with an average value of −17.45 kJ/g (−471.55 kJ/C-mol) [55] being used in the following. This value can be confirmed using the elemental composition of cellulose (C_6_H_10_O_5_)_n_ and Equation (1), which yields −16.1 kJ/g.

For the calculation of the metabolic heat yield coefficient *Y_Q/X_* as a function of the biomass yield coefficient (*Y_X/S_ = 1/Y_S/X_*), the growth equation (Equation (3)) and the respective enthalpy balance was required. Here, the carbon source was described by CH_nH_O_nO_. For the fungal biomass, the composition of *Aspergillus niger* (C_1_H_1.6_O_0.55_N_0.12_; [56]) was assumed.

(3) $$\begin{array}{ll} Y_{S/X}{\mathrm{CH}}_{n_{H}}O_{n_{O}}+ & 0.12\;{\mathrm{NH}}_{3}+Y_{O/S}\;O_{2}\\ & \to \;{\mathrm{CH}}_{1.6}O_{0.55}N_{0.12}+Y_{C/S}\cdot {\mathrm{CO}}_{2}+Y_{H/S}\cdot H_{2}O \end{array}$$

The enthalpy balance can be simplified according to Equation (4), considering that the combustion enthalpies of CO_2 (g)_, H_2_O_(l)_, and O_2(g)_ are zero. For simplicity, the protonation of CO_2_ to ${\mathrm{HCO}}_{3}^{-}$ is neglected. The resulting equation is:

(4) $$\Delta_{r}H_{X}=Y_{Q/X}=-\left(\Delta_{C}H_{X}-Y_{S/X}\Delta_{C}H_{S}-0.12\;\cdot \Delta_{C}H_{NH3}\right).$$

**Figure 4:**
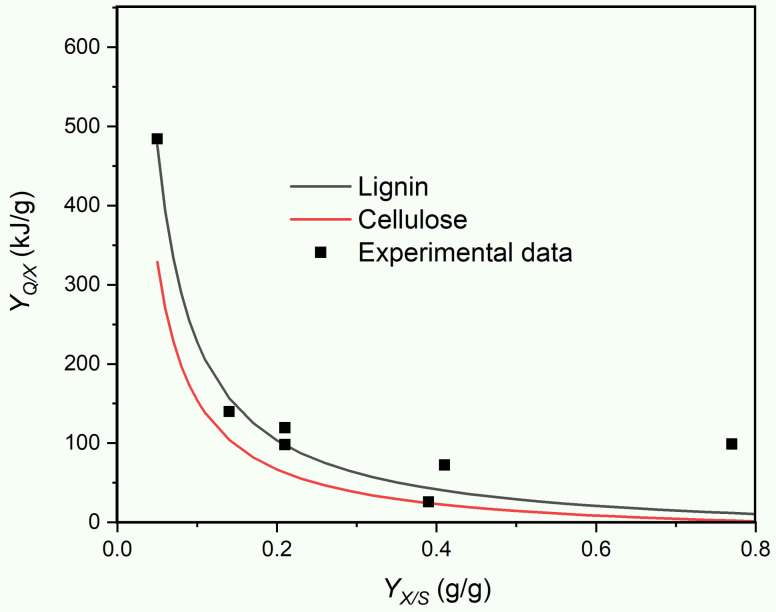
Metabolic heat yield coefficient (*Y_Q/X_*) as a function of the biomass yield coefficient (*Y_X/S_*) for the seven investigated fungi. The solid lines represent the thermodynamically allowed state space, under the assumption that only one substrate was used for growth and no other products have formed besides biomass. The symbols correspond to the experimentally determined parameters derived from fungal growth on wheat straw (Table 2) and represent the means of the triplicate cultures (the corresponding standard deviations can be retrieved from Table 2).

$\Delta_{C}H_{X}$, $\Delta_{C}H_{S}$, and $\Delta_{C}H_{NH3}$ are the combustion enthalpies of biomass, substrate, and ammonia dissolved in water (−295.6 kJ/mol) [57], respectively. Equation (4) allows us to estimate the heat release as a function of the biomass yield coefficient *Y_X/S_*. Figure 4 shows the comparisons of the measured metabolic heat yield coefficients *Y_Q/X_* with the results of the calculations based on Equation (4), assuming that either lignin or cellulose is the only substrate utilised. To keep simple and consistent with the literature, the absolute value of the *Y_Q/X_* values is given, although it is clear that almost all growth reactions are exothermic; consequently, the *Y_Q/X_* has a negative symbol. From the comparison of the data in Table 2 and Figure 4, it can be concluded that: (i) the relationship of heat release, in terms of between *Y_Q/X_* and *Y_X/S_* is well described by the thermodynamic model (Equation (4)); (ii) the given accuracy of the analytics does not allow the distinguishing between the utilisation of lignin or cellulose; and (iii) the determination of *Y_X/S_* is much more error-prone than that of *Y_Q/X_*, as can be inferred from the respective standard deviations of these parameters that are listed in Table 2. The extraordinarily high degree of inaccuracy of the *Y_X/S_* obtained for *P. chrysogenum* (Table 2) is a particularly striking example for the last observation.

The *Y_Q/X_* values shown in Table 2 were used to convert the cumulative metabolic heat signals into time courses of the fungal biomass (i.e., the increases as compared with the respective starting points) as indicated by the right y-axes of Figure 3B,D,F,H,J (not shown for *S. chlorohalonata* and *S. rugosoannulata* because the underlying data has already been published before [2]). These fungal biomass time courses served as the determination of the apparent maximum growth rates (*AMGR*) of the investigated fungal strains, which are also compiled in Table 2, as follows: First, derivatives of the metabolic heat-based biomass yield-over-time data were derived with the help of the ‘differentiate’ function of the OriginPro 2020 software. The respective highest values were then identified by performing descriptive column statistics in OriginPro 2020, which were considered as the *AMGR* (Table 2). Moreover, the respective cultivation times were extracted from the first derivatives of time courses of the fungal biomass data mentioned before, until the onset of fungal growth at *AMGR* (herein denoted as *t_AMGR_*), and are also listed in Table 2. Finally, a parameter denoted as competitive growth potential (*CGP*) was calculated as the quotient of the *AMGR* and *t_AMGR_* for all fungal strains, respectively, which is additionally compiled into Table 2.

### 3.3. Correlation between Y_Q/X_ Values and Other Fungal Growth-Related Parameters

With respect to the parameters inherently reflecting the relationship between resource channeling into biomass production and other functional parameters, the *Y_Q/X_* values may be more reliable and meaningful than *Y_X/S_* data for the following reasons: Firstly, they seemingly are more robust and less prone to errors than the *Y_X/S_* values that were determined for the fungal growth on the complex solid substrate applied within the present study (Table 2). Secondly, the *Y_X/S_* values are usually calculated based on the disappearance of the presumable growth substrate, assuming that the fungal biocatalytic activity is the only cause for the substrate’s disappearance. In this respect, the measurement of the metabolic heat fluxes provides a more direct proof for biochemical substrate conversion due to fungal activity.

**Figure 5:**
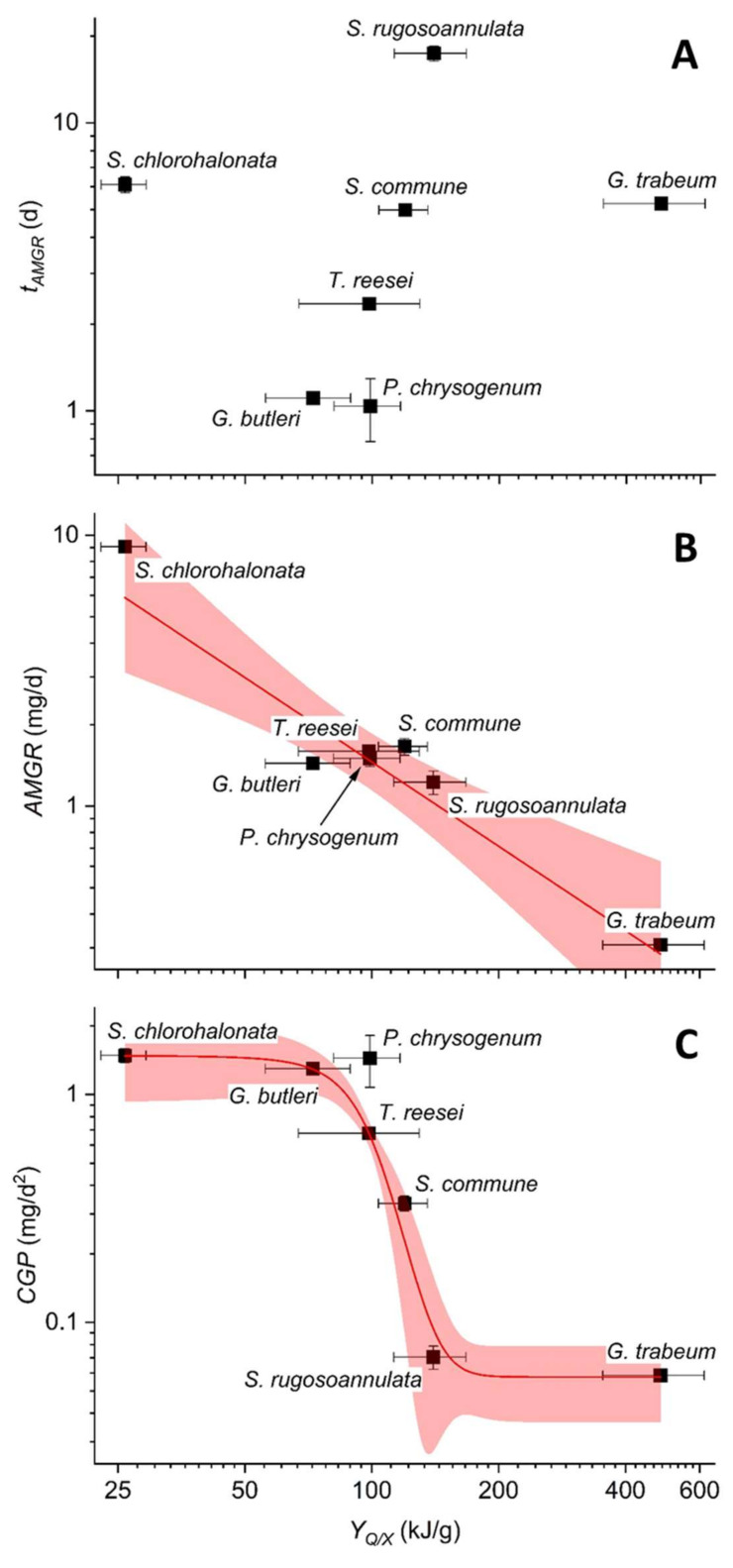
(**A**) The cultivation time until the fungal growth at a maximal rate was observed (*t_AMGR_*), (**B**) the apparent maximum growth rate (*AMGR*), and (**C**) the competitive growth potential (*CGP*) as functions of the metabolic heat yield coefficient (*Y_Q/X_*) for the 7 investigated fungi, respectively. Logarithmic scaling was chosen to facilitate the reading. The symbols correspond to experimentally determined parameters derived from fungal growth on wheat straw (Table 2) and represent the means ± standard deviations (calculated according to the Gaussian error propagation rules) for the triplicate cultures. The solid red lines in (**B**,**C**) result from the linear (coefficient of determination *R*^2^ > 0.87) and non-linear fitting of the experimentally determined data by employing a dose-response model (*R*^2^ > 0.99), respectively; the corresponding 95% confidence bands are labelled in pink.

Consequently, we have assessed possible correlations between the *Y_Q/X_* values obtained for the investigated fungal strains and other parameters, of which these correlations are especially indicative of the potential fungal abilities to grow on the solid lignocellulosic substrate. No obvious relation between the *t_AMGR_* and *Y_Q/X_* values could be observed (Figure 5A). However, a fairly good correlation between the *AMGR* and the corresponding *Y_Q/X_* values was obtained upon the application of a simple linear fit model (Figure 5B). Nevertheless, apart from *S. chlorohalonata* and *G. trabeum* forming the respective cornerstones, all other investigated fungi clustered together in this type of analysis. By contrast, an excellent resolution of all investigated fungal strains and a very high correlation in the double-logarithmic *CGP* vs. *Y_Q/X_* plots was achieved by applying a non-linear dose-response fit model (coefficient of determination *R***^2^** > 0.99) (Figure 5C). In this context, it must be noted that the models applied for data fitting have no underlying conceptual value. They have simply been employed as quantitative descriptors of the potential relationships between the investigated parameters.

## 4. Discussion

Following the current views advocating the application of trait-based approaches in fungal ecology [1,7,9,13,14,15], the present study attempted to qualify the non-invasive measurement of metabolic heat fluxes via biocalorimetry techniques for the quantification and possible refinement of growth-related traits that characterise free-living saprotrophic lignocellulose decomposer fungi. Fungal growth (or biomass accumulation) rates are considered to represent “true traits” contributing together with other true traits to so-called “trait complexes”, describing the performance of an individual in relation to co-occurring taxa [8]. For instance, fungal growth rates may influence complexes of effect traits, determining how organisms affect their environment (such as carbon/nutrient mineralisation or the formation of soil organic matter), and also response trait complexes governing organismic responses to different conditions, such as their combativeness or moisture stress tolerance [8]. Our biocalorimetric approach allowed for the non-invasive monitoring of fungal growth over time (Figure 3) and the straightforward determination of corresponding descriptive apparent parameters, such as the *AMGR* and *t_AMGR_* values (Table 2). In actuality, the quantification of the growth processes of filamentous fungi on solid substrates is not trivial due to the spatial and temporal inhomogeneities brought about by the interplay between substrate characteristics and the typically polar tip growth [58,59,60]; which usually hampers representative sampling required for invasive biomass determination methods, and to clearly distinguish between different growth phases. Fungal growth monitoring by conventional means is challenging even in liquid cultures, where growth of many filamentous fungi in the form of pellets can complicate optical cell density determinations and representative sampling for further biomass analysis methods, and result in overlaps of different growth phases at the same time due to progressively increasing nutrient and oxygen limitations towards the centres of mycelial pellets [61,62]. In this respect, biocalorimetric growth monitoring can provide a convenient alternative to the conventional biomass determination methods not only for solid-state but also for liquid cultivation systems. Similarly, other non-invasive monitoring methods, such as online respirometry have also been suggested as valuable tools for the characterization of fungal growth processes and related physiological states in bioreactor batch culture experiments [63].

In our work, we abstained from using biocalorimetrically-obtained growth curves (Figure 3B,D,F,H,J) for fungal growth modelling, delimitation of different fungal growth phases, or determination of lag phases as has been frequently addressed by other studies [60,62,63,64]. Instead, we aimed at simple growth parameters that could be derived easily and are robust enough to describe the efficiency of the investigated fungi with respect to utilisation of the applied lignocellulosic substrate for growth. Here, *AMGR* (Table 2) denotes the apparent maximum growth rate without discriminating between potentially overlapping growth phases. The cultivation time until growth at the maximal rate is observed (*t_AMGR_*: Table 2) does not delimitate the lag and the exponential growth phase as otherwise usual [60,63], and hence integrates both phases. We refer to the quotient formed from the *AMGR* and *t_AMGR_* as competitive growth potential (*CGP*: Table 2), which takes both the apparent maximum fungal growth rate as well as the time span until its onset into account. Hereby, the *CGP* inherently involves the potential fungal capacity for a combination of exploitation (competition for nutrients through resource depletion) and interference competition (i.e., the more that biomass is formed, the more space is occupied; thereby spatially limiting access of competitors to the resource and/or inhibits them), which cannot clearly be distinguished from each other in decomposer fungi [5,8,65]. The discussed growth-related parameters seem to be suitable for linking the potential of fungi for biomass production with the corresponding ecological strategy in resource utilisation. They thus represent life history traits, which reflect the resource investment of a species into fitness components, such as growth, reproduction, and survival [9,14,66]. In terms of fungal life history strategies, rapid growth is typically associated with an R (ruderal)-selected strategy [1,5]. Fungi possessing C (combative)-selected characteristics grow sometimes slowly, whereas S (stress-tolerant) strategists do not display particularly slow growth [5].

In addition to the biocalorimetric analysis of fungal growth kinetics (Figure 3B,D,F,H,J) and determination of parameters describing the temporal dimension of fungal growth (Table 2), we were in particular addressing potential trait parameters being suitable to describe the degree of resource investment into fungal biomass versus further functional attributes, which hereby may concomitantly indicate different ecological strategies followed by the investigated fungi [2]. Potentially, increased *Y_X/S_* and decreased *Y_Q/X_* values would both go along with a predominant resource channelling into biomass formation, whereas increasing additional investments into further energetically costly functional attributes aiming at e.g., the extraction of nutrients from recalcitrant substrates through lignin degradation, reproduction via spore formation, or antagonism brought about the production of antibiotics [5,8,9,67] would progressively decrease the *Y_X/S_* and increase the *Y_Q/X_* values. This tendency is confirmed by the *Y_Q/X_* and *Y_X/S_* values that were determined in the present study (Table 2), which fall within the range of previously published data for fungal organisms [68,69,70,71] and considerably vary depending on the respective fungus investigated. For instance, a prominent resource investment into growth is consistently indicated by comparatively high *Y_X/S_* and low *Y_Q/X_* values for the fast-growing moulds *G. butleri*, *P. chrysogenum*, and *S. chlorohalonata*, respectively (Table 2). In contrast, substantial resource channelling into further functional attributes is suggested by the lowest and second lowest *Y_X/S_* values observed with the comparatively slow-growing basidiomycetes *G. trabeum* and *S. rugosoannulata*, respectively, concomitantly matching with the highest and second highest *Y_Q/X_* values obtained (Table 2). Representing a brown-rot and a white-rot fungus, respectively, *G. trabeum* and *S. rugosoannulata* occupy special ecological niches through their abilities to attack particularly recalcitrant constituents of lignocellulose (crystalline cellulose in the case of brown-rot, lignin in the case of white-rot) with the help of resource-demanding extracellular machineries [2,4,22,23]. Nevertheless, a shortcoming we found was that the *Y_X/S_* values determined in our study were associated with considerable errors (with standard deviations ranging from about 10 to more than 200% of the respective means as could be deduced from the data shown in Table 2), which is owed to the limited accuracy of the underlying gravimetric determinations, particularly in cases of the only low substrate dry mass losses observed with *P. chryogenum* and *G. butleri* (Figure 2, Table S1). Here, *Y_Q/X_* values could provide a much more reliable and obviously less error-prone alternative, where the corresponding standard deviations were found to range between approximately 12 and about 32% of the respective means as could be estimated from Table 2.

A closer look at the possible correlations between *Y_Q/X_* and the parameters *AMGR*, *t_AMGR_*, and *CGP* suggests that the latter is strongly linked with *Y_Q/X_* (Figure 5C). Potentially, a high *CGP* value indicates fast-growing strains, thus pointing to a high fungal potential for primary resource capture typically associated with an R-selected life history strategy [5]. The strains *S. chlorohalonata*, *G. butleri*, and *P. chrysogenum* all display high and essentially comparable *CGP* values, and they separate along the lower range of the *Y_Q/X_* axis (Figure 5C), basically indicating an extensive resource channelling into growth along with accompanying investments into other functionalities at varying degrees. These fungi are also well known to reproduce via extensive spore production, and are known as producers of especially (hemi)cellulolytic enzymes and diverse metabolites (Table 1; please also refer to Section 1). Increasing preferences for C- or S-selected life history strategies are suggested along with progressively declining *CGP* and increasing *Y_Q/X_* values (Figure 5C). For instance, decay fungi employing a C-selected life history sometimes grow only slowly and can utilise more recalcitrant substrate structures than R strategists [5]. In particular, the characteristics of *S. rugosoannulata* and *G. trabeum* already mentioned before (Figure 5C, Table 2) are in line with such a behaviour. Nevertheless, in accordance with variable resource channelling between growth and further functional attributes indicated by variable *Y_Q/X_* values (Figure 5C), fungal taxa may not strictly be assigned to a specific life history strategy, while their behavior in a particular respect can be defined by such terms [5]. Fungi also often combine characteristics from different life history strategies, which form continuous scales rather than delimited cornerstones [5,8].

## 5. Conclusions

Our study demonstrates that non-invasive biocalorimetric analysis can successfully be employed to determine different growth-related fungal activity parameters, such as apparent maximum growth rates (*AMGR*), cultivation times until the onset of fungal growth at the *AMGR* is observed (*t_AMGR_*), quotients formed from the *AMGR* and *t_AMGR_* (referred to as the competitive growth potential, *CGP*), and heat yield coefficients (*Y_Q/X_*) that indicate the degree of resource investment into fungal biomass versus other functional attributes. These parameters seem suitable for linking fungal potentials for biomass production to corresponding ecological strategies employed during resource utilisation, and therefore may be considered as fungal life history traits. A close connection exists between the *CGP* and *Y_Q/X_* values, which suggests an interpretation in terms of fungal life history strategies. While the feasibility of the approach of our study was demonstrated using single fungal species in a standardised laboratory environment, it still needs to be validated within a community context [1,8].

## Figures and Tables

**Table 1:** Overview of the fungal strains employed in the present study.

Fungal Strain	Phylogeny (Phylum, Class, Order)	Characteristics	References
*Gloeophyllum trabeum* Persoon: Fries Murrill (DSM 1398)	Basidiomycota, Agaricomycetes, Gloeophyllales	Causes the brown-rot decay of wood	[4,22]
*Gongronella butleri* (Lendner) Peyronel & Dal Vesco (DSM 2917)	Mucoromycota, Mucoromycetes, Mucorales	Potential production of enzymes, metabolites, lipids, and chitosan in lignocellulose biorefineries	[41,42,43]
*Penicillium chrysogenum* Thom (DSM 848)	Ascomycota, Eurotiomycetes, Eurotiales	Production of cellulases and hemicellulases during lignocellulose degradation; penicillin (antibiotic) production	[35,37,38]
*Schizophyllum commune* Fries (DSM 11223)	Basidiomycota, Agaricomycetes, Agaricales	Intermediate between brown-rot and white-rot fungi, with potential applicability for lignocellulosic feedstock saccharification purposes	[23,24]
*Stachybotrys chlorohalonata* Andersen and Trane strain A-2008-2 (DSM 27588)	Ascomycota, Sordariomycetes, Hypocreales	Environmentally ubiquitous mould with preference for cellulose-rich substrates	[2,31]
*Stropharia rugosoannulata* Farlow ex Murrill (DSM 11372)	Basidiomycota, Agaricomycetes, Agaricales	Litter-decaying white-rot fungus	[2,44]
*Trichoderma reesei* Simmons (DSM 769)	Ascomycota, Sordariomycetes, Hypocreales	Outstanding cellulase producer, e.g., in lignocellulose biorefineries	[25,26,27]

**Table 2:** The observed biomass yield coefficients (*Y_X/S_)*, metabolic heat yield coefficients (*Y_Q/X_*), apparent maximum growth rates (*AMGR*), and cultivation times until growth at maximal rate, respectively (*t_AMGR_*); the competitive growth potential values (*CGP*) of fungal wheat straw cultures during the 32 (*S. chlorohalonata* and *S. rugosoannulata*) or 20 days of cultivation (all other fungi) ^a^.

Fungus	*Y_X/S_* (g/g)	*Y_Q/X_* (kJ/g)	*AMGR* (mg/d) ^b^	*t_AMGR_* (d) ^c^	*CGP* (mg/d^2^) ^b^
*G. trabeum*	0.05 ± 0.02	484.3 ± 131.0	0.31 ± 0.01	5.25 ± 0.04	0.06 ± 0.00
*G. butleri*	0.41 ± 0.57	72.4 ± 16.5	1.44 ± 0.03	1.11 ± 0.02	1.30 ± 0.03
*P. chrysogenum*	0.77 ± 1.58	99.0 ± 17.8	1.50 ± 0.10	1.04 ± 0.26	1.45 ± 0.37
*S. commune*	0.21 ± 0.11	119.6 ± 15.9	1.66 ± 0.12	4.98 ± 0.13	0.33 ± 0.03
*S. chlorohalonata*	0.39 ± 0.04 ^c^	26.0 ± 3.2 ^c^	9.07 ± 0.32	6.11 ± 0.38	1.48 ± 0.11
*S. rugosoannulata*	0.14 ± 0.03 ^c^	140.0 ± 27.2 ^c^	1.23 ± 0.12	17.40 ± 1.04	0.07 ± 0.01
*T. reesei*	0.21 ± 0.11	98.3 ± 31.3	1.59 ± 0.02	2.36 ± 0.02	0.68 ± 0.01

^a^ Values represent the means ± standard deviations (calculated according to the Gaussian error propagation rules) for triplicate cultures (means ± absolute deviations from the duplicate cultures for the *AMGR*, *t_AMGR_*, and *CGP* values of *G. butleri* and *T. reesei*, respectively, where the outliers have been identified using a Dean–Dixon test and were excluded from further analysis). ^b^ The *CGP* was calculated as *AMGR* divided by *t_AMGR_*, respectively. ^c^ Data derived from reference [2].

## Data Availability

The data that support the findings of this study are available from the corresponding authors.

## Supplementary Materials

The following supporting information can be downloaded at: https://www.mdpi.com/article/10.3390/microorganisms10081675/s1, Table S1: Total dry masses, substrate dry mass losses, total lignin contents, and lignin losses of the fungal wheat straw cultures. The means and standard deviations (SD) from the triplicate cultures are shown, respectively. Substrate dry masses were calculated as the differences between total dry masses (Table S1) and fungal biomasses (Table S2), respectively (data not shown). Mass losses were always calculated as the difference between the corresponding initial and final amounts, respectively, and are expressed in terms of absolute (mg) and relative (i.e., in relation to the corresponding initial amount) values, respectively. Table S2: Total sugars of the solids remaining after aqueous extraction, total water-extractable sugars, sum of total sugars, losses of total sugars, fungal biomasses, and increases in fungal biomasses in fungal wheat straw cultures. The means and standard deviations (SD) from the triplicate cultures are shown, respectively. The mass losses in the sum of both sugar fractions and fungal biomass increases were calculated as the difference between the corresponding initial and final amount, respectively, and are expressed in terms of absolute (mg) and relative (i.e., in relation to the corresponding initial amount; for sugar losses only) values, respectively.
