# Supplementary material for: Fungal Lignocellulose Utilisation Strategies from a Bioenergetic Perspective: Quantification of Related Functional Traits Using Biocalorimetry
Source: Microorganisms. 2022 Aug 19;10(8):1675. doi: 10.3390/microorganisms10081675 (PMC9415514; doi:10.3390/microorganisms10081675)
Supplement: Supplementary file 1 [file microorganisms-10-01675-s001.zip › microorganisms-1840702-supplementary.pdf]

Supplementary Material

Fungal Lignocellulose Utilisation Strategies from a Bioenergetic Perspective: Quantification of Related Functional Traits Using Biocalorimetry

Hieu Linh Duong [1,2], Sven Paufler [1], Hauke Harms [1], Dietmar Schlosser [1,*], and Thomas Maskow [1,+]

[1] Department of Environmental Microbiology, Helmholtz-Centre for Environmental Research - UFZ, Permoserstraβe 15, 04318 Leipzig, Germany

[2] Vietnamese-German University (VGU), Le Lai Street, Hoa Phu Ward, Thu Dau Mot City, Binh Duong Province, Vietnam

Content

Table S1……….. 2

Table S2……….. 4

* Correspondence: thomas.maskow@ufz.de (T.M.); dietmar.schlosser@ufz.de (D.S.)

Table S1. Total dry masses, substrate dry mass losses, total lignin contents, and lignin losses of fungal wheat straw cultures. Means and standard deviations (SD) from triplicate cultures are shown, respectively. Substrate dry masses were calculated as differences between total dry masses (Table S1) and fungal biomasses (Table S2), respectively (data not shown). Mass losses were always calculated as the difference between the corresponding initial and final amount, respectively, and are expressed in terms of absolute (mg) and relative (i.e. in relation to the corresponding initial amount) values, respectively.

Fungal strain	Total dry mass (mg)		Substrate dry mass loss (mg)		Substrate dry mass loss (%)		Total lignin (mg)		Lignin loss (mg)		Lignin loss (%)	
	Mean	SD	Mean	SD[a]	Mean	SD[a]	Mean	SD	Mean	SD[a]	Mean	SD[a]
Initial value[b]	558.29	47.83	NA[c]		NA[c]		74.00	6.32	NA[c]		NA[c]	
S. commune DSM 11223[d]	484.34	13.68	91.89	49.77	16.55	9.08	75.78	1.90	ND[e]		ND[e]	
G. butleri DSM 2917[d]	555.17	34.67	18.03	59.13	3.25	10.65	77.36	4.39	ND[e]		ND[e]	
Initial value[f]	561.06	20.84	NA[c]		NA[c]		74.46	2.75	NA[c]		NA[c]	
G. trabeum DSM 1398[d]	498.89	27.79	65.72	34.75	11.76	6.24	82.04	5.28	ND[e]		ND[e]	
T. reesei DSM 769[d]	502.43	24.10	69.75	32.04	12.49	5.75	76.25	8.47	ND[e]		ND[e]	
P. chrysogenum DSM 848[d]	561.97	4.72	6.56	21.40	1.17	3.83	76.61	5.79	ND[e]		ND[e]	
Initial value[g]	521.41	14.85	NA[c]		NA[c]		74.89	11.81	NA[c]		NA[c]	
S. rugosoannulata DSM 11372[h]	390.85	22.53	149.02	27.21	28.68	5.30	42.69	5.86	32.19	13.18	42.99	18.86
Initial value[i]	492.33	2.60	NA[c]		NA[c]		70.68	3.75	NA[c]		NA[c]	
S. chlorohalonata A-2008-2 (DSM 27588)[h]	312.12	12.23	286.51	15.31	58.50	3.14	55.63	1.43	15.05	4.01	21.30	5.78

[a] Calculated according to Gaussian error propagation rules.

[b] Initial values for *S. commune* and *G. butleri* cultures.

[c] Not applicable.

[d] Values correspond to a cultivation time of 20 days.

[e] Not detectable.

[f] Initial values for *G. trabeum*, *T. reesei*, and *P. chrysogenum* cultures.

[g] Initial values for S*. rugosoannulata* cultures.

[h] Values correspond to a cultivation time of 32 days.

[i] Initial values for *S. chlorohalonata* cultures.

Table S2. Total sugars of solids after aqueous extraction, total water-extractable sugars, sums of total sugars, losses of total sugars, fungal biomasses, and increases in fungal biomasses in fungal wheat straw cultures. Means and standard deviations (SD) from triplicate cultures are shown, respectively. Mass losses in the sum of both sugar fractions and fungal biomass increases were calculated as the difference between the corresponding initial and final amount, respectively, and are expressed in terms of absolute (mg) and relative values (i.e. in relation to the corresponding initial amount; for sugar losses only), respectively.

Fungal strain	Total sugars of solids after aqueous extraction (mg)		Total water-extractable sugars (mg)		Sum of total sugars (mg)		Loss of total sugars as based on the sum (mg)		Loss of total sugars as based on the sum (%)		Fungal biomass (mg)		Fungal biomass increase (mg)	
	Mean	SD	Mean	SD	Mean	SD[a]	Mean	SD[a]	Mean	SD[a]	Mean	SD	Mean	SD[a]
Initial value[b]	481.2	41.1	26.3	2.3	507.5	41.1	NA[c]		NA[c]		3.1	0.6	NA[c]	
S. commune DSM 11223[d]	387.5	10.4	36.0	2.7	423.5	10.8	84.0	42.5	16.6	8.5	21.1	1.4	17.9	1.6
G. butleri DSM 2917[d]	459.8	28.0	11.6	1.1	471.4	28.1	36.1	49.8	7.1	9.8	18.0	2.6	14.9	2.7
Initial value[e]	484.1	17.9	16.9	2.1	501.0	18.0	NA[c]		NA[c]		2.5	0.3	NA[c]	
G. trabeum DSM 1398[d]	410.8	25.3	11.5	1.5	422.3	25.4	78.7	31.1	15.7	6.2	6.0	0.9	3.5	0.9
T. reesei DSM 769[d]	412.6	13.1	34.9	0.2	447.4	13.1	53.6	22.2	10.7	4.5	13.6	3.3	11.1	3.3
P. chrysogenum DSM 848[d]	475.4	7.9	15.9	1.6	491.3	8.1	9.7	19.7	1.9	3.9	9.9	1.1	7.5	1.1
Initial value[f]	444.7	4.7	33.7	5.0	478.4	6.8	NA[c]		NA[c]		1.8	0.3	NA[c]	
S. rugosoannulata DSM 11372[g]	327.9	20.0	18.0	0.6	345.9	20.0	132.5	21.1	27.7	4.4	20.2	3.5	18.5	3.5
Control[h]	419.1	4.9	30.1	2.4	449.2	5.5	NA[c]		NA[c]		2.6	0.6	NA[c]	
S. chlorohalonata A-2008-2 (DSM 27588)[g]	150.5	13.1	29.5	0.1	180.0	13.1	269.2	14.2	59.9	3.2	108.9	8.8	106.3	8.8

[a] Calculated according to Gaussian error propagation rules.

[b] Initial values for *S. commune* and *G. butleri* cultures.

[c] Not applicable.

[d] Values correspond to a cultivation time of 20 days.

[e] Initial values for *G. trabeum*, *T. reesei*, and *P. chrysogenum* cultures.

[f] Initial values for S*. rugosoannulata* cultures.

[g] Values correspond to a cultivation time of 32 days.

[h] Initial values for *S. chlorohalonata* cultures.
